# Traumatic lung laceration secondary to avulsed lung adhesion - A case report

**DOI:** 10.1016/j.tcr.2023.100862

**Published:** 2023-06-04

**Authors:** Ahmed F. Ramzee, Zeenat Bakhsh, Ruben Peralta, Sandro Rizoli, Ayman El-Menyar, Hassan Al-Thani, Talat Chughtai

**Affiliations:** a Department of Surgery, Trauma Surgery, Hamad Medical Corporation, Doha, Qatar; b Trauma and Vascular Surgery, Clinical Research, Hamad Medical Corporation, Doha, Qatar; c Clinical medicine, Weill Cornell Medical college, Doha, Qatar; d Department of Surgery, Qatar University, Hamad Medical Corporation, Doha, Qatar

**Keywords:** Pulmonary laceration, Avulsion force, Intrathoracic injuries, Hemothorax, Trauma

## Abstract

**Background:**

Pulmonary lacerations caused by an avulsion force on an adhesion between the lung and chest wall following blunt thoracic injury are very rare. They may result in pneumothorax and/or hemothorax and may not be immediately apparent clinically or radiologically.

**Case presentation:**

We present the case of a healthy 34-year-old male who sustained blunt thoracic injury. He was clinically stable, and his initial routine images were unremarkable. The patient was discharged home on the same day. He presented a week later with a massive hemothorax requiring surgical intervention which revealed bleeding from an avulsed adhesion between the lung and chest wall. Bleeding was successfully controlled by hemostatic agent, and the patient had an uneventful recovery.

**Conclusion:**

Hemothorax requiring intervention from an avulsed adhesion may occur following blunt thoracic trauma. Initial imaging and clinical finding may be misleading. Close follow up and adequate patient education should be ensured prior to discharge following seemingly trivial trauma.

## Introduction

Pulmonary lacerations are tears in the pulmonary parenchyma which can occur following penetrating or blunt chest trauma [1,2]. Lacerations due to previously formed lung adhesions are rare and may not be obvious on initial presentation, but may lead to complications such as pneumothorax, as well as massive hemothorax [2]. In this report, we describe a case of massive hemothorax due to lung laceration from a traumatic avulsed lung adhesion that was initially unrecognized.

## Case report

A 34-year-old male was brought to the emergency department (ED) after being hit by a vehicle while riding his bicycle. Upon arrival, he was vitally stable and his primary and secondary survey were unremarkable. His only complaint was right sided chest discomfort. Extended Focused Assessment with Sonography in Trauma (EFAST) was negative, and his initial chest x-ray was normal. After a brief stay in the ED, his symptoms improved, and he was discharged home on the same day, with instructions to return if he appreciated any chest symptoms.

**Fig. 1:**
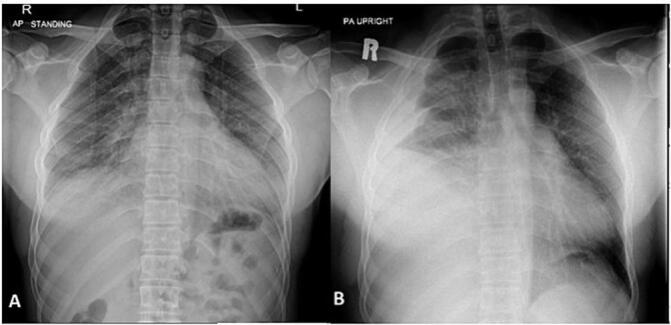
A - chest X-ray on initial admission. B – Chest X-ray on subsequent admission showing right sided effusion.

**Fig. 2:**
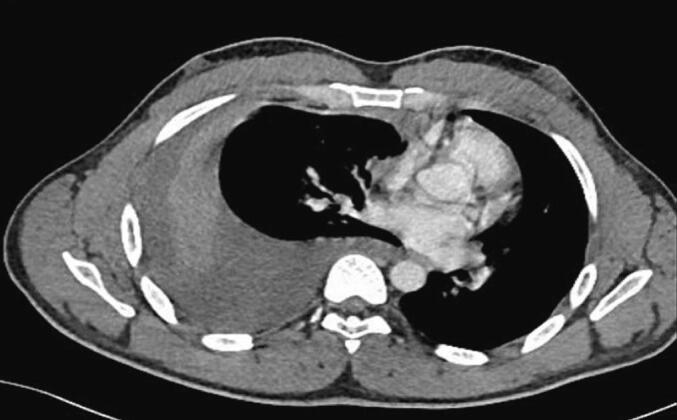
CT scan of the thorax demonstrating a massive right sided hemothorax with shift of the mediastinum to the left.

**Fig. 3:**
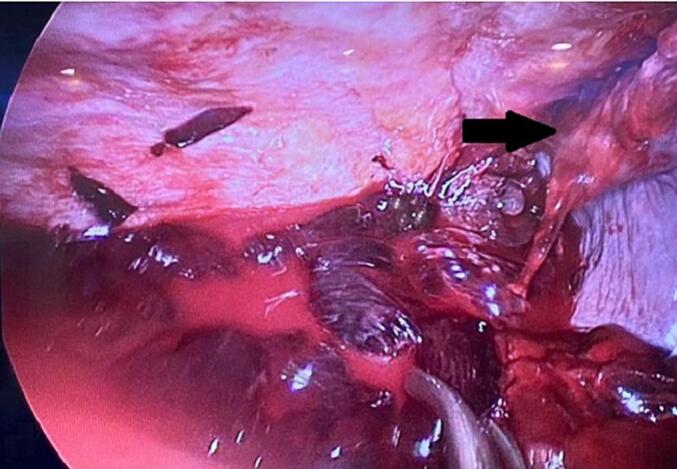
Thoracoscopic view showing large amounts of clots as well as the adhesion (black arrow).

**Fig. 4:**
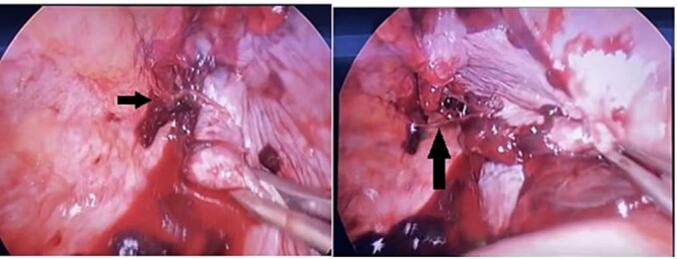
Avulsed adhesion between pleura and upper lobe (Adhesion marked with black arrows).

**Fig. 5:**
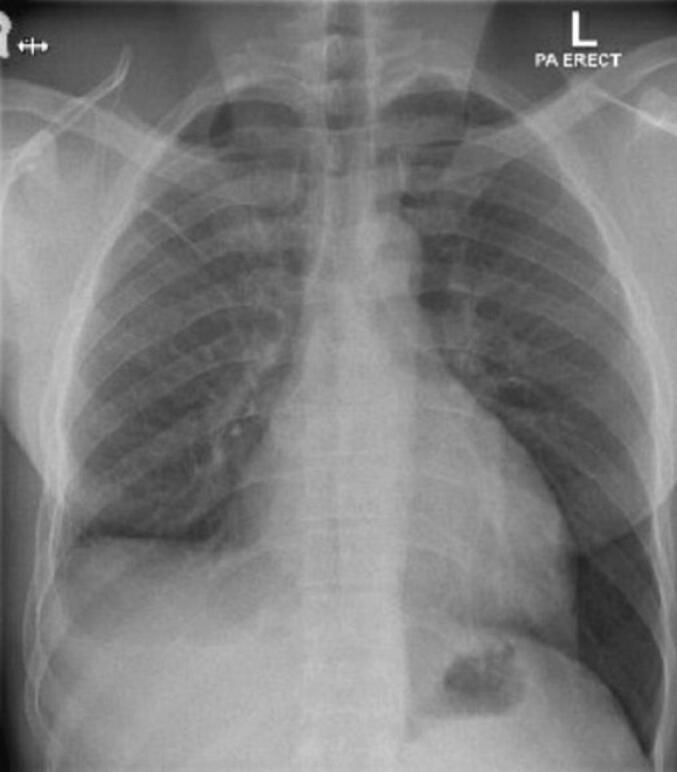
Chest X-ray on follow up 1 week later.

One week later, the patient presented to the trauma room complaining of right sided chest pain with breathing and upon movement. He was tachypneic and his examination was remarkable for right sided chest tenderness and decreased air entry in the right basal zone of the lungs. A chest x-ray revealed a large right sided pleural effusion, without any obvious rib fractures. Fig. 1 compares the initial and subsequent chest x-ray. Since his initial presentation one week previously, he had a 3 g/dl drop in the hemoglobin level. As the patient was otherwise stable, he underwent a computed tomography (CT) scan of the thorax which revealed a massive right sided effusion of high density suggestive of blood, along with a mediastinal shift to the left (Fig. 2). There was no evidence of lung contusion or rib fractures. Given these findings, a 28 French intercostal tube was inserted, which drained 500 ml of dark blood. He was admitted to the TICU (Trauma Intensive Care Unit) for observation, and over the course of the day, the total chest tube output was nearly 1000 ml with a persistent large residual effusion on follow up chest x-ray. Due to the persistent large volume output, as well as retained hemothorax, we decided to proceed with video assisted thoracoscopic surgery (VATS). Surgery revealed a large volume of clots in the right chest cavity with a small area of the upper lobe of the lung partially avulsed from an adhesion to the right parietal pleura (Fig. 3, Fig. 4). There was no active bleeding at the time and there were no other injuries noted. Topical absorbable hemostatic agents, in the form of oxidized cellulose (Surgical®, Ethicon) mesh and snow/powder, were placed over the avulsed portion of the chest wall and lung and a 32 French chest tube was placed. There was no air leak noted at the end of surgery. The patient post-operative course was uneventful, the chest drain was removed on day 3 post-operative, and he was discharged on day four. Of note, the patient did not have any prior history of lung disease, including any recent known infections. A follow up chest X-ray a week later showed a clear chest with no evidence of a hemothorax (Fig. 5). He was followed up for one month after his surgery and was doing well with no active issues. Pleural fluid that was sent for cytology did not reveal any evidence of malignancy.

## Discussion

Pulmonary lacerations are tears in the parenchyma of the lung and are a part of a wide spectrum of thoracic injuries. Although commonly occurring with penetrating injury, they are also seen after blunt trauma as the parietal pleural fibrous adhesions to the lung are very well vascularized [1,2]. High velocity injuries such as motor vehicle crashes and falls from a height pose an increased risk of such parenchymal injuries [1]. Lacerations can result in a hematoma, pneumatocele, pneumothorax, hemothorax or hemopneumothorax depending on the site and extent of injury [1,2]. These injuries occur rarely in isolation and carry a risk of mortality and morbidity, especially when associated with massive bleeding, other extra-thoracic injuries, and morbidity due to operative intervention [1]. Fractured and internally displaced ribs may directly penetrate the lung and cause a laceration [1].

In blunt trauma, nearly 50 % of lung lacerations may not be clinically apparent on initial chest x-rays due to associated consolidation that may occur with adjacent lung contusions [3]. CT scan imaging has proven to be more sensitive and specific in identifying these injuries [2]. The findings on CT scan reflect the elastic recoil of the lung causing the damaged lung to retract away from the laceration forming a round or oval cavity, filled with air, blood or both, rather than forming a linear defect as seen in other solid organs [1,2].

Wagner classified pulmonary lacerations into 4 types based on CT pattern, mechanism of injury, location of associated rib fracture, or surgical findings [1]. Type 4 is the rarest type that occurs due to rupture of previously formed adhesions between the pleura and the lung after a violent shearing force is applied. The diagnosis of this type of injury is usually made during surgery or on autopsy. Our patient whose initial examinations were normal, presented a week later with a massive hemothorax, indicating that the initial avulsion may have only resulted in a very small amount of bleeding which accumulated over time, eventually resulting in his symptoms. CT scan did not reveal any obvious findings of lung parenchymal injury apart from a massive hemothorax and the diagnosis was revealed only during surgery.

Only a small proportion of patients with thoracic trauma require surgical intervention and from these, only one third would require lung resection to control bleeding or removal of severely injured tissue [3]. The main non-emergent indication for an intervention would be a retained large hemothorax which can act as a medium of infection or lead to a chronic fibrothorax. The main principle of surgery in these patients is to assess the extent of injury, control active bleeding and drainage of hemothorax [4]. Accepted Advanced Trauma Life Support guidelines for emergent thoracotomy include acute blood loss over 1500 ml or recorded ongoing drainage of more than 200–400 ml over 2 to 4 h [5]. Recently thoracoscopic surgery (VATS) has gained popularity in managing stable patients with thoracic injuries with its added advantage of better visualization of the pleural cavity apart from being minimally invasive. Chou reported significantly decreased rates of post-traumatic infection, length of ventilatory dependency and overall hospital stay with VATS rather than tube thoracostomy alone [6]. In our patient the main indication for surgical intervention was the persistent retained hemothorax despite tube thoracostomy with continued drainage of blood. Although no active bleeding was found, the intervention allowed us to perform a thorough washout of the pleural cavity with removal of large clots and to identify the cause of the bleeding. Chou also recommended early VATS for those who fail to clear their pleural cavities within 24–72 h following tube thoracostomy despite all non-surgical interventions as this improved outcome as described previously and that delayed intervention beyond 5–7 days maybe associated with increased morbidity due to adhesion formation [6,7]. Intra pleural fibrinolysis via a chest tube has shown to be effective in the management on empyema and complicated parapneumonic effusions, however its role in retained hemothorax is not fully validated as of now [8]. A small, randomized control trial of 35 patients showed no difference in outcomes when compared to VATS [9]. This may be a feasible alternative to surgical intervention and warrants larger studies.

## Conclusion

Lung laceration are not uncommon injuries following high velocity blunt chest trauma and need to be suspected in patients with hemothorax. Type 4 injuries are the rarest form of injuries and may not have obvious evidence of lung injury even on CT scan. Close follow up of patients with seemingly trivial findings post high velocity injuries to the chest is essential to ensure there is no delayed complications. VATS is a feasible option in hemodynamically stable patients with retained or newly developed hemothorax even within a week of injury.

## CRediT authorship contribution statement

All authors contributed to the study design, the analysis and interpretation of data, and manuscript writing and approved the final manuscript.

## Funding

None.

## Financial disclosure

None.

## Ethics approval and consent to participate

This case report was approved with a waiver of informed consent by the medical research center at Hamad Medical Corporation (HMC), Doha, Qatar (IRB# MRC-04-22-659) on 19/10/2022 as data were collected anonymously with no identifiable images and retrospectively.

## Availability of data and material

All data were presented in the manuscript and tables.

## Declaration of competing interest

All the authors have no conflict of interest and financial issue to disclose.

## References

[1] Miller D.L., Mansour K.A. (2007 Feb). Blunt traumatic lung injuries. Thorac. Surg. Clin..

[2] Thoongsuwan N., Kanne J.P., Stern E.J. (2005 May). Spectrum of blunt chest injuries. J. Thorac. Imaging.

[3] Carboni Bisso I., Gemelli N.A., Barrios C., Las Heras M. (2021). Pulmonary laceration. Trauma Case Rep..

[4] Lodhia Joshil Vinod (2019). Video-assisted thoracoscopic surgery in trauma: pros and cons. J. Thorac. Dis..

[5] ATLS Subcommittee, American College of Surgeons’ Committee on Trauma, International ATLS working group (2013). Advanced trauma life support (ATLS®): the ninth edition. J. Trauma Acute Care Surg..

[6] Chou Y.P., Kuo L.C., Soo K.M. (2014). The role of repairing lung lacerations during video-assisted thoracoscopic surgery evacuations for retained haemothorax caused by blunt chest trauma. Eur. J. Cardiothorac. Surg..

[7] Chou Yi-Pin (2015). Video-assisted thoracoscopic surgery for retained hemothorax in blunt chest trauma. Curr. Opin. Pulm. Med..

[8] Foo C.T., Herre J. (2021 Jun). Use of intrapleural fibrinolytic therapy in a trapped lung following acute traumatic haemothorax. Case Rep. Pulmonol..

[9] Kumar S., Rathi V., Rattan A., Chaudhary S., Agarwal N. (2015 Sep). VATS versus intrapleural streptokinase: a prospective, randomized, controlled clinical trial for optimum treatment of post-traumatic residual hemothorax. Injury.

